# The Role of a Smart Health Ecosystem in Transforming the Management of Chronic Health Conditions

**DOI:** 10.2196/44265

**Published:** 2023-12-18

**Authors:** Rebecca Nourse, Tilman Dingler, Jaimon Kelly, Dominika Kwasnicka, Ralph Maddison

**Affiliations:** 1 School of Exercise and Nutrition Sciences Deakin University Burwood Australia; 2 School of Computing and Information Systems University of Melbourne Melbourne Australia; 3 Centre for Health Services Research Faculty of Medicine The University of Queensland Brisbane Australia; 4 NHMRC CRE in Digital Technology to Transform Chronic Disease Outcomes Melbourne School of Population and Global Health University of Melbourne Melbourne Australia; 5 Faculty of Psychology SWPS University of Social Sciences and Humanities Wroclaw Poland

**Keywords:** smart home, health, chronic condition, chronic illness, digital health, technology, behavior change, wearable, smart technology, smart health, economic, cost, security, data storage, implementation

## Abstract

The effective management of chronic conditions requires an approach that promotes a shift in care from the clinic to the home, improves the efficiency of health care systems, and benefits all users irrespective of their needs and preferences. Digital health can provide a solution to this challenge, and in this paper, we provide our vision for a smart health ecosystem. A smart health ecosystem leverages the interoperability of digital health technologies and advancements in big data and artificial intelligence for data collection and analysis and the provision of support. We envisage that this approach will allow a comprehensive picture of health, personalization, and tailoring of behavioral and clinical support; drive theoretical advancements; and empower people to manage their own health with support from health care professionals. We illustrate the concept with 2 use cases and discuss topics for further consideration and research, concluding with a message to encourage people with chronic conditions, their caregivers, health care professionals, policy and decision makers, and technology experts to join their efforts and work toward adopting a smart health ecosystem.

## The Problem: Impact and Management of Chronic Conditions

The prevalence of chronic conditions, such as cardiovascular disease, stroke, chronic obstructive pulmonary disease, and diabetes, and the proportion of people with more than 1 health condition, multimorbidity, are rising [1,2]. People with chronic conditions have complex needs and require long-term management and prevention support, but health care systems that provide siloed services do not support this [3]. Personal health data (eg, blood pressure and heart rate) used to inform treatment are generally captured on an episodic basis in a clinical context, leading to a fragmented picture of a person’s health status [4]. Additionally, there are many barriers to self-care [5], but it is considered integral for people with chronic conditions to improve health-related quality of life, reduce the risk of developing additional health conditions and complications, and reduce strain on health care systems [6-8]. Self-care is defined as the participation in the maintenance, monitoring, and management of behaviors during daily life to support health [9]. At a personal level, reported barriers to self-care include a lack of knowledge and skills, the experience of symptoms, and medication side effects, to name a few [10]. Nevertheless, responsibility does not solely sit here. The effective management of chronic conditions requires an approach that considers personal, behavioral, social, and infrastructural factors to promote health and foster the efficiency of future health care systems.

## Toward a Solution: Digital Health

**Figure 1:**
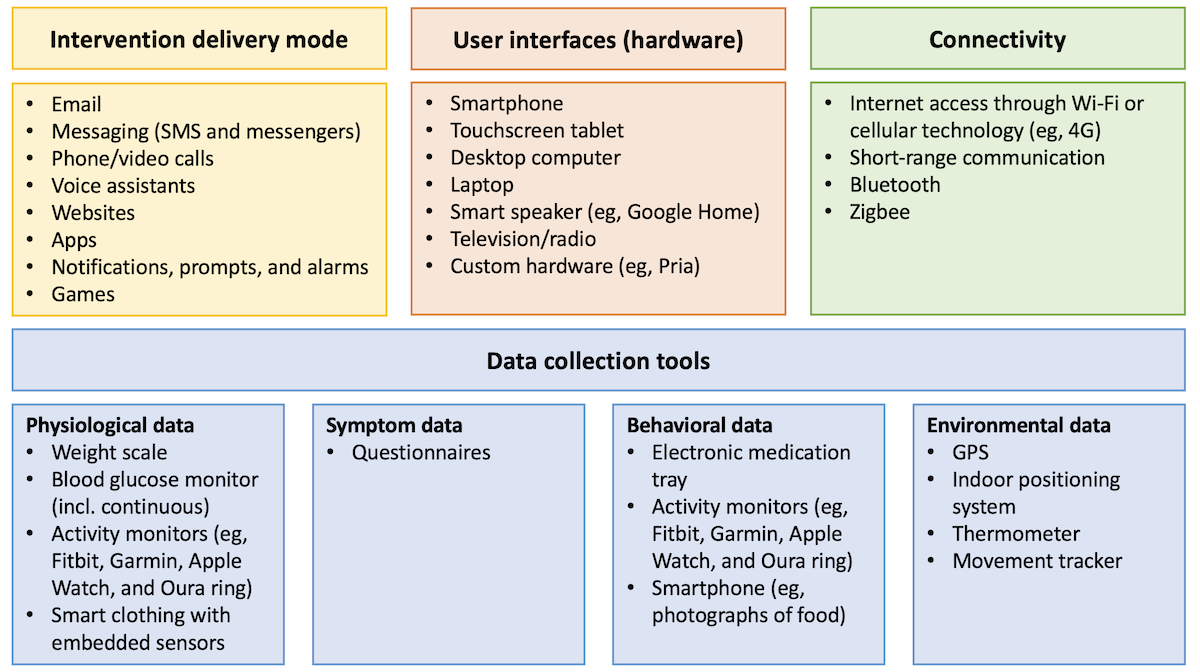
Examples of technologies employed in digital health interventions.

Digital health refers to the use of technologies, including information and communications technology, the internet, and pervasive sensing, to transform health systems and health care delivery [11,12]. Within the health care system, telemonitoring and telemedicine provide remote diagnostics and treatments, electronic medical records hold patient data, and algorithms support clinicians with evidence-based decisions [13]. Advanced data processing, such as activity recognition using classifiers powered by machine learning, allow large amounts of health data to be processed in real-time to make sense of people’s lifestyle and activity levels. Researchers have also implemented a range of digital health tools and solutions to support self-care behavior in people with chronic conditions [14-18], typically including a user interface for interacting with the intervention, data collection tools, education, and support based on user data. Examples of technologies used in research-based digital health interventions are presented in Figure 1.

Over the past decade, there has also been an exponential increase in the self-driven use of digital health by people with chronic conditions [19]. Supply and demand have driven this proliferation; technologies are increasingly available and accessible, and people can use them independently without needing support from a health care professional [20]. In 2022 there were 52,565 health care and medical apps on the Google Play Store [21] and 51,370 on the Apple App Store [22]. Between 2016 and 2019, the number of wearable devices, such as smart watches, rose by 400 million [23].

While digital health is here to stay and offers countless opportunities, the landscape is disjointed: health care systems have not adopted technologies at the same rate as society, interventions developed by academics do not make it out into the real world, and people can easily collect their own health data but often lack medical knowledge to use it effectively for health improvement [24]. Health care can be transformed by joining up efforts to leverage digital health, including the interoperability of technologies and advancements in big data and artificial intelligence. A smart health ecosystem is 1 solution to facilitate a coordinated approach to health care while supporting people to manage their health outside of clinical settings. This article describes our vision for a smart health ecosystem, illustrating the concept through 2 use case studies. We also reflect on key considerations and provide recommendations for further research.

## A Smart Health Ecosystem

### Overview

**Figure 2:**
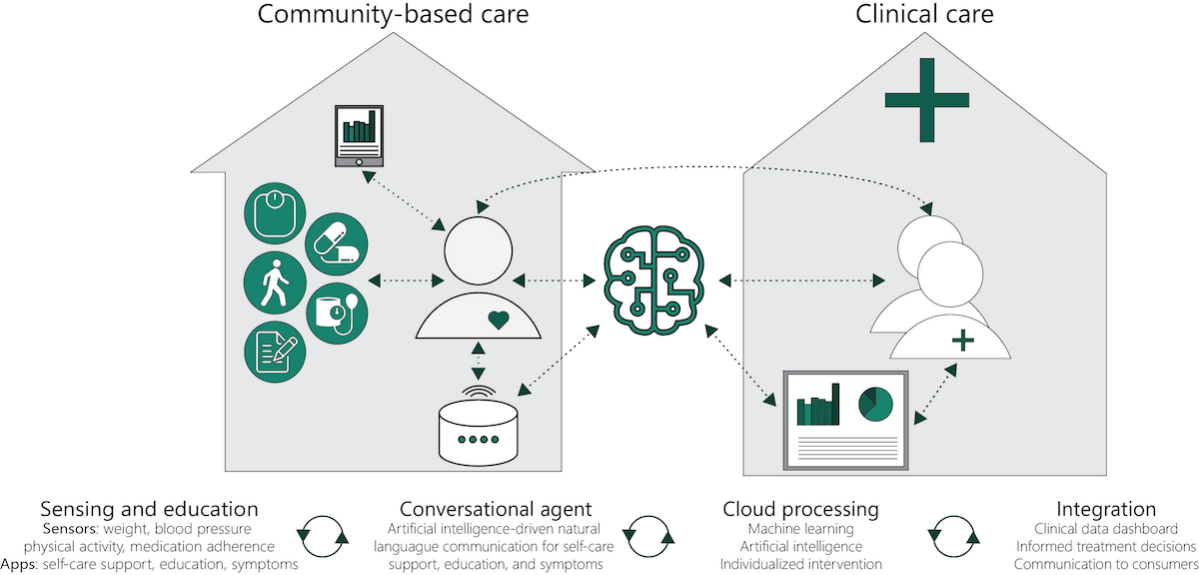
A smart health ecosystem.

Our vision for a smart health ecosystem comprises sensing, processing, and communications technologies (Figure 2). These technologies unobtrusively and continuously collect data about an individual’s health status and context, allowing for a long-term picture of health and well-being. The analysis of these data allows access to comprehensible data summaries, personalized behavioral support (eg, alarms, notifications, and advice), and connection to health care professionals and other sources of support (eg, family, friends, or caregivers) when required.

### Opportunities

A smart health ecosystem may benefit people with chronic conditions and the health care system. Here we give an overview of these opportunities.

#### A Comprehensive Picture of Health in Context

Integrating continuously collected data from multiple sources and processing with sophisticated algorithms within a smart ecosystem allows for a comprehensive picture of health and related contextual factors.

Through the internet of things, a networked system of wireless connected devices, there are now more ways to passively (without user input) collect data in real time without having to visit a health care setting [25]. Off-the-shelf devices (eg, wearables and smartphones) can measure physiological and behavioral features such as weight, nutrition, physical activity, blood pressure, medication adherence, and temperature [25,26]. The environmental context can also be measured, providing additional opportunities to tailor interventions. For example, by determining the user’s location and the weather forecast, we might suggest alternatives to going outside for a walk when it is raining and prompt medication taking while the user is home, where it is likely that they will have access to their medication. This integration enables the development of health care solutions with intelligent prediction capabilities and offers means to provide personalized behavioral support [27,28]. Use case 1 gives an example of this in action.

There are limitations to using self-reported data, which are notoriously under- (dietary intake) or overreported (physical activity) [29-31]. Yet, it is still important to obtain self-reported data to build this comprehensive picture of health status. Here, technology can help too. Ecological momentary assessment (EMA) permits real-time self-reporting of behavior and experiences [32]. EMA, facilitated by mobiles and tablets, has been used to assess affect, social environment, physical activity, cognition, and physical environment [33].

Digital phenotyping, which uses passively sensed data from multiple sources to allow for a moment-by-moment quantification of behavior, also showcases the potential of data [34,35]. This method can enhance the diagnosis and treatment of chronic conditions through earlier detection of condition onset, relapse, or treatment response. An example of this is provided in use case 2. Moreover, comprehensive data collection would be particularly beneficial for knowledge generation and intervention development in lesser investigated behaviors (often due to difficulties in obtaining accurate measurements) such as sleep, stress reduction, and symptom management [36].

#### Personalization and Tailoring

A smart health ecosystem is a flexible approach that can be personalized on several levels; we have already mentioned the personalization of advice enabled by collecting and analyzing an individual’s data. The content and devices within the system may also be tailored depending on, for example, a person’s experience with technology, diagnosis, personality, and physical and cognitive capabilities. A range of end user interfaces, including websites, apps, SMS text messages, email, conversational agents, virtual reality, and web-based chat, can provide a mode to view meaningful data and access personalized interventions from anywhere, at any time [37].

#### Theoretical Advancements

Theory helps describe and understand problems and guide the development of solutions to address them [38]. It is recommended that theories be used to guide intervention development and implementation as this can improve the acceptability and increase the effectiveness of interventions [39]. Not only can a smart health ecosystem be informed by theory, but it can also provide an opportunity for testing and advancing theory through the generation and processing of ecologically valid and real-time data. This may lead to new lenses through which we can view phenomena and identify additional opportunities for change, further driving the theory development cycle.

#### Health Care System

Digital health is already changing the balance of power between health care professionals and end users [11]. By bringing together state-of-the-art solutions, a smart health ecosystem offers new possibilities to deliver more person-centered, flexible, and responsive health care to manage and prevent chronic conditions [40]. First, health care professionals will better understand the people they care for, allowing them to provide optimal treatment and facilitate shared decision-making. Second, sharing health data with preferred health care professionals, whose experience is difficult to replace with technology, reassures users and promotes trust. Finally, by presenting data in an accessible format and providing tailored, evidence-based recommendations, a smart health ecosystem can empower the user to make informed decisions and take timely action without needing to visit a clinic.

#### Large Language Models

Advancements in large language models (LLMs), such as Generative Pretrained Transformers, could revolutionize digital health. While the safety, reliability, acceptability, and effectiveness of LLMs require investigation, they can help realize the potential of a truly smart health ecosystem. Opportunities for LLMs in the management of chronic conditions include the following:

- Supporting remote management and telehealth by analyzing data and providing real-time insights.
- Assisting in clinical decision-making and research by analyzing clinical guidelines and literature, and to provide recommendations for action.
- Facilitating communication between health care professionals and patients who speak different languages by translating speech and text.
- Enhancing health literacy and patient education by providing access to information, explaining concepts, and answering questions.
- Generating personalized support based on preferences and data from electronic health records, wearable devices, and patient-reported measures.
- Scaling health services by automating self-assessments, check-ins, and guiding patients to relevant health services.

## Vision and Use Cases

To illustrate this vision for a smart ecosystem to support people with chronic conditions, we present 2 potential use cases and relevant scenarios.

### Use Case 1: Type 2 Diabetes

Matilda is 43 years old, lives with her 2 young children, and works full-time. She recently saw her general practitioner (GP) for low energy levels and stress. Her GP ran some tests and found that Matilda had an elevated fasting blood glucose level of 6.2 mmol/L and a BMI of 31 kg/m^2^. In addition, Matilda reported that her mother had type 2 diabetes. Following tests, Matilda is diagnosed with type 2 diabetes and is told by her GP that she will need to take insulin as part of her management plan. She is also encouraged to make changes to her diet and increase her physical activity levels. Matilda is busy, leading her to skip meals and snack late at night once her children have gone to bed to ease her stress. She does not have time, or energy, to cook meals after her long days at work but wants to improve her health so she can be there for her children. Matilda liked the idea of personalized support and enrolled in the Smart Health Ecosystem.

Matilda was given access to a continuous glucose monitor (CGM), a smartphone app, a wrist-worn fitness tracker, and a set of Bluetooth weight scales. The CGM provides blood glucose readings every 5 minutes (288 per day), allowing Matilda to track her blood glucose levels and trends. Previous research has shown that self-monitoring of blood glucose is an integral component of effective treatment planning for many patients with diabetes taking insulin [41,42]. The fitness tracker collects data on her activity levels, heart rate, sleep time, and location of her daily activities. Matilda was happy that she could use her personal smartphone to access the app as she was not keen to carry another device around. When Matilda first signed into the app, she was asked questions to assess her personality profile, likes, and dislikes, and was prompted to set some goals; the subsequent choice of behavioral support was based on this information. Matilda feels motivated when she receives regular feedback, likes to eat Thai food, enjoys dancing, and has a competitive nature. She chose to set goals for weight loss, waist circumference, and physical activity levels. Using the app, Matilda can monitor her blood glucose levels to make informed decisions about food choices and physical activity in relation to her goals. She weighs herself once a week using the smart scales, and she can also input her waist circumference into the app. Progress toward her waist circumference and weight loss targets is congratulated with “points” for a competition in the app; Matilda tops the leaderboard most weeks and wants to stay there. The app also sends Matilda reminders of her progress throughout the week, motivating her to continue making positive lifestyle changes.

Over time, the system learns Matilda’s routine. Matilda usually goes food shopping on a Saturday afternoon. Using her location data, when Matilda is on her way to the supermarket, she gets a notification on her phone that prompts her to open her app for a personalized shopping list. The list contains options for quick-to-prepare recipes and ready-prepared products based on her dietary needs and weight loss target. When the system identifies that Matilda has some free time—based on her location and activity status, she receives prompts to try a web-based Zumba class—provided through the app. During the class, her heart rate is transmitted to the app; depending on her heart rate, she gets on-screen prompts to increase her effort. As her fitness increases, algorithms adjust her targets and activity difficulty.

Matilda permitted the app to send summary-level data to be sent to her GP. Her GP can review these data to see her progress and facilitate discussion at her next visit or initiate a referral to a dietitian for follow-up. Similarly, once referred to a dietitian, these data could be summarized and provided to the dietician to generate a more personalized care plan. The Smart Health Ecosystem allows Matilda to be an active participant in her own care and reduce the risk of health complications and additional clinic appointments.

### Use Case 2: Heart Failure

Herbert is 71 years old, lives alone at home, and has heart failure. Over 1 week, Herbert’s symptoms worsened with increased shortness of breath, a cough, fatigue, and swollen feet; as a result, his granddaughter suggested that he go to the hospital. Herbert was admitted for 3 nights, during which time he was monitored closely and treated with diuretics to reduce his fluid retention. He felt much better after his treatment and was enrolled in the Smart Health Ecosystem prior to being discharged.

Once home, Herbert was encouraged to measure his weight daily using Bluetooth scales. If a weight measurement was not detected by midday, and if motion sensors detected that he was at home, Herbert would receive a notification on his smartphone reminding him to step on the scale. One day, the Smart Health Ecosystem detected that Herbert’s weight had increased by 3 kg and that he had not accessed his sensor-enabled medication box in the days prior; it is likely that he had not taken his medication and was consequently experiencing fluid retention. An activity tracker indicated that Herbert was sitting longer than usual, prompting a symptom questionnaire delivered and answered through a conversational agent (eg, Alexa). Herbert reported that he was again experiencing shortness of breath and was feeling tired. System algorithms were used to analyze these parameters in relation to Herbert’s standard measurements (collected by EMA multiple times a day) and prompted an intervention.

The conversational agent prompted Herbert to contact his GP within 24 hours and reminded him to take his medication. An alert with summary-level data was sent to his heart failure nurse specialist for review. Due to the event’s proximity to Herbert’s recent hospital admission, the nurse called Herbert via the system to check in and advised him to adjust his diuretic medication. Subsequent hospital readmission was avoided, resulting in reduced hospital costs and increased quality of life for Herbert.

## Considerations for Developing and Implementing a Smart Health Ecosystem

The following section synopsizes key areas that warrant consideration and further research before integrating a smart health ecosystem into practice. A summary of this discussion and our key messages are provided in Textbox 1.

**Summary of key messages for consideration and further research for developing, implementing, and adopting a smart health ecosystem.**

**System design:** Methodological research on agile and dynamic intervention development that considers user needs and contexts, as well as clinical evidence and social support mechanisms.

**Technical requirements:** More testing of devices, especially in the target populations to ensure validity, reliability, and acceptability.

**Data security:** Comply with national and international standards to ensure data security and apply privacy-by-design solutions where possible.

**Data storage:** Cheap cloud storage enables large-scale data collection while conveniently leaving it up to the storage provider to back up and secure the data.

**System implementation:** Consider implementation during intervention development, including ways the system can integrate into clinical practice and end users’ existing routines, preferences, and skill sets. Transdisciplinary theories can be adopted for this purpose.

**Economic costs and evaluation:** Understand economic costs and benefits by exploring modular approaches to intervention components and content and answering questions about the financial impacts on the broader health and social system.

**Health equity:** Natural user interfaces allow populations who tend to be tech-unsavvy access to health services. Additionally, effective technology use has the potential to scale health services and, as a result, lower their costs, which makes them affordable to disadvantaged populations.

### System Design

Implementing a smart health ecosystem will only be possible when the approach is collectively accepted and adopted by consumers, clinicians, allied health care workers (eg, nurses, physiotherapists, and social workers), and other health care stakeholders [43]. People have varying perspectives on technology, often stemming from their past experience and the design and usability of devices [44]. User experiences are varied across, for example, different personalities, diagnoses, and physical capabilities; hence, features may be acceptable to 1 person and not another [45]. Likewise, features that are acceptable to an individual at one time, or place, may not be acceptable at another time or location.

Multimorbidity can significantly impact the design and effectiveness of smart health ecosystems due to the complexity of managing multiple health conditions. People with multimorbidity risk being bombarded with multiple alerts and overwhelmed by messages from their various health care professionals. A smart health ecosystem can support care coordination by facilitating seamless communication and information sharing among different health care professionals. LLMs can help tailor care plans for the individual’s specific needs and help schedule multiple appointments. However, participatory or co-design values users as “experts” of their own experience and can be used to ensure that a smart health ecosystem meets user needs and preferences [46]. To help meet user needs, agile science, an iterative and adaptive development approach, can be used to evaluate the incremental and iterative development of digital health programs through initial development, optimization, and open source “release” phases. Toward the development of adaptive, responsive, and individualized programs, agile science also involves cocreation with end users and health care professionals and using machine learning and artificial intelligence principles [47].

### Technical Requirements

Passively collected data on internet of things devices are subject to inaccurate and inconsistent measurements and missing data [48]. Some technologies are still emerging and require stabilization, and the validity and accuracy of devices continue to be tested [49-51]. Further appraisal of the validity and accuracy of devices in people with chronic conditions is also required. Devices should be carefully selected; a framework for device selection that considers technical, user experience, health and digital health literacy, data, regulations, safety and privacy, and investment concerns is available to support this [52]. To provide access to safe and evidence-based digital health products (eg, wearables sensors and CGM), we need a strong regulatory environment for digital health products. This will also improve the commercial viability of new innovations. Smart health ecosystems will need to align with existing regulations and be nimble enough to pivot as the regulatory environment inevitably changes in the future.

### Data Security

The acceptability of a smart ecosystem and its constituent components is closely linked to security, privacy, and trust [53]. Such systems need to be robust and resilient for end users (eg, patients) and clinicians to feel confident in their everyday use. Technology infrastructure bears the risk of cyberattacks [54] and exploitation of inappropriately collected personal and sensitive data. This potential for breach is significant in the presence of networked sensors and digital devices, which make up a smart ecosystem. This is primarily due to the reliance on wireless communications (which makes eavesdropping more straightforward) and low-energy technologies (which are unable to implement complex security protocols on their own) [25]. Such risks, however, can be reduced by applying privacy-by-design principles, such as storing and processing data on edge devices only (without the need to send it over a communication network).

Data security issues must be fully addressed and comply with national and international standards or regulations such as the HIPAA (Health Insurance Portability and Accountability Act) in the United States and the General Data Protection Regulation, applicable to the European Union and European Economic Area. Further, the Medical Device Regulation is a regulation that sets out requirements for the safety and performance of medical devices, including certain digital health technologies. It defines rules for classifying and certifying medical devices, including software used for medical purposes.

### Accountability

To ensure data accountability, clear guidelines on storage, control, and ownership are essential. As a rule of thumb, only minimal personal data should be collected and stored. Data aggregates and the principle of k-anonymity can be used to mitigate risks of data breaches and help advance research by releasing such anonymous data sets to the public [55]. An issue of note here concerns how information is handled when it falls outside the scope of HIPAA regulations, for example, when a consumer uses third-party health apps or web portals to access health data for personal use [56]. These health apps are often hosted by external entities and may subsequently share user data with limited transparency and accountability. Importantly, most of these apps are not subject to HIPAA regulations and may share user activity data with other third parties involved in advertising and profiling [56]. A practical approach to adopt is a policy that clearly details a data host’s obligation to protect and secure stored data and their liability for any unauthorized access or data breaches. Similarly, individuals can take responsibility for who they give consent to use their data. It is crucial to recognize that sharing data across different jurisdictions and countries presents complexities as international hosts and suppliers may not adhere to the same regulatory frameworks [25]. Furthermore, blockchain technologies have the potential to tightly control and document data access; the ownership model and transparency of blockchain solutions allow patients to determine who can view data and any views and modifications are trackable on a public ledger [57].

### Data Storage

In recent years, the cost of data storage has significantly decreased. Various software companies offer cheap cloud storage while guaranteeing high-speed data access and taking care of backups and security. However, centralized data storage can introduce bottlenecks and especially network latency when it comes to retrieving and storing data from consumer devices. A shift to decentralized storage methods, also called edge computing, can alleviate some of these issues as data is stored and processed locally without needing a network to communicate to a central server. In either case, data storage is now abundant and rich user data can be collected continuously. Parallel processing and ever more efficient algorithms allow us to make sense of this “big data” and use it to find patterns, monitor patient states, and inform diagnostics.

### System Implementation

A smart health ecosystem should seamlessly integrate technologies into people’s everyday lives and routines; this is likely to lead to greater adoption and engagement. Theories such as normalization process theory [58], or social practice theory [59,60], provide lenses through which we can understand how interventions might build on, integrate with, or disrupt everyday life. Implementation approaches must prevent digital exclusion and inequities in health care access [61,62]. A key benefit of a smart health ecosystem is that it can provide more than just telemonitoring. Training on how to use the system may be required [45]; this training could be provided by the system itself by adapting to the users’ learning targets over time. Furthermore, by providing feedback on measurements and the consequences of actions taken by the user, the system can reinforce learning—promoting future independence. From the health care perspective, a stepped approach to rollout might allow for natural adaptation without disrupting standard practice.

From the health care perspective, the smart health ecosystem will need to work alongside existing patient management and electronic health care record systems. Moreover, we need to create care management approaches and infrastructure that allow practitioners to engage with the smart health system without increasing the burden. For example, a busy GP does not have time scheduled in their daily routine to follow up or track alerts that can be generated by devices worn or used by patients. The smart health system will need to seamlessly interact with patient management systems across the continuum of care to enable health care professionals to make informed, data-driven decisions without leaving their existing clinical workflows. Alternatively, we may have to restructure the way we deliver health care to adapt to the use of smart health systems. For example, nurse-led interventions are being used to support people to self-care at home [63]. Nurses or other allied health professionals may be well-suited to ensure case management via the smart health ecosystem. Many GPs also believe that business and financial pressures in general practice influence their choice of consultation mode and where their time is allocated for non–patient-facing activities [64]. Therefore, there is more work to be done around the best funding models to support the effective use and monitoring of smart health care as also a top priority for primary care and health policy. A defined reimbursement framework for smart health ecosystems will be needed to overcome barriers to commercialization and uptake by health care professionals and consumers. Moreover, appropriate reimbursement will be an important step to ensure equity of access, particularly for those most in need. A stepped approach to rollout might allow for natural adaptation without disrupting standard practice. The new era of digital health can shift administrative work to technology, but the transition needs to be carefully designed. Economic costs and evaluation

Health care resources are scarce; thus, many systems have focused on mechanisms that optimize the efficiencies and long-term sustainability of the health system. Understanding the economic costs and the potential gains for the patient, clinician, and health care system is vital. Smart health care may be cost-saving to individuals and the health system, but economic evaluations are yet to confirm this. We know that web-based models of care are cost-saving over time, ranging from immediate to over 9 years [65]. However, the ways in which a smart health ecosystem will generate cost savings may be more nuanced, and our understanding of these continues to evolve [66].

Future research is needed to understand whether a smart health ecosystem can reduce hospital presentations and admissions. Furthermore, we need future research to understand whether smart health care can reduce patient travel and parking costs and ever-growing waiting times to see health professionals, and sick days will help clarify the potential economic gains of a smart health ecosystem approach. To promote cost efficiency, we suggest using a modular approach whereby the user interface, sensors, and content can be added, removed, or modified depending on user needs and preferences.

### Health Equity

The use of digital health systems, tools, and interventions is reshaping health care services; however, this is likely disproportionate to health needs. Evidence suggests that individuals residing in economically disadvantaged areas, vulnerable and remote communities with varied cultural and linguistic backgrounds, people with disabilities, and those with limited health and digital health literacy are less likely to access or benefit from digital health services [67]. On the contrary, vulnerable population groups stand to gain the most from these services. Natural user interfaces, such as voice assistants, lower the barriers of entry to health services for vulnerable populations, and the ability to scale health services using technologies, such as LLMs, increases their accessibility. While these population groups used to bear a disproportionate burden of health care needs and encounter additional barriers in accessing essential medical services, technologies can grant them both access to and provide services at lower costs. Acknowledging and addressing the digital divide through constant reconsideration is imperative in order to enhance equity in accessing telehealth services [67].

## Conclusions

By leveraging existing digital health technologies and integrating theory- and evidence-based behavioral support, a smart health ecosystem has the potential to transform the reactive health care model into a proactive model that will meet user needs. There are considerable challenges around data security and storage, technology access, and adoption, but these will likely be addressed at the current transformation rate. We urge people with chronic conditions, their caregivers, health care professionals, policy and decision makers, and technology experts to join their efforts in developing, implementing, and evaluating digital health technologies to accelerate the adoption of a smart health ecosystem.

## Abbreviations

CGM: continuous glucose monitor
EMA: ecological momentary assessment
GP: general practitioner
HIPAA: Health Insurance Portability and Accountability Act
LLM: large language model
